# Cost-effectiveness in extracorporeal life support in critically ill adults in the Netherlands

**DOI:** 10.1186/s12913-018-2964-6

**Published:** 2018-03-09

**Authors:** Annemieke Oude Lansink-Hartgring, Dinis Dos Reis Miranda, Dirk W. Donker, Jacinta J. Maas, Thijs Delnoij, Marijn Kuijpers, Judith van den Brule, Erik Scholten, Hendrik Endeman, Alexander P. J. Vlaar, Walter M. van den Bergh, On behalf of the Dutch ECLS study group

**Affiliations:** 1 University Medical Center Groningen, University of Groningen, PO box 30.001, 9700 RB Groningen, the Netherlands; 2 Erasmus Medical Center Rotterdam, Rotterdam, the Netherlands; 3 University Medical Center Utrecht, Utrecht, the Netherlands; 4 Leiden University Medical Center, Leiden, the Netherlands; 5 Maastricht University Medical Center, Maastricht, the Netherlands; 6 Isala, Zwolle, the Netherlands; 7 Radboud University Medical Center, Nijmegen, the Netherlands; 8 Sint. Antonius Ziekenhuis Nieuwegein, Nieuwegein, the Netherlands; 9 Onze Lieve Vrouwen Gasthuis, Amsterdam, the Netherlands; 10 Amsterdam Medical Center, Amsterdam, the Netherlands

**Keywords:** Extracorporeal life support, Cost-effectiveness, Critical care, Intensive care unit, Outcome, Quality of life

## Abstract

**Background:**

Extracorporeal life support (ECLS) is used to support the cardiorespiratory function in case of severe cardiac and/or respiratory failure in critically ill patients. According to the ELSO guidelines ECLS should be considered when estimated mortality risk approximates 80%. ECLS seems an efficient therapy in terms of survival benefit, but no undisputed evidence is delivered yet. The aim of the study is to assess the health-related quality of life after ECLS treatment and its cost effectiveness.

**Methods:**

We will perform a prospective observational cohort study. All adult patients who receive ECLS in the participating centers will be included. Exclusion criteria are patients in whom the ECLS is only used to bridge a procedure (like a high risk percutaneous coronary intervention or surgery) or the absence of informed consent. Data collection includes patient characteristics and data specific for ECLS treatment. Severity of illness and mortality risk is measured as precisely as possible using measurements for the appropriate age group and organ failure. For analyses on survival patients will act as their own control as we compare the actual survival with the estimated mortality on initiation of ECLS if conservative treatment would have been continued. Survivors are asked to complete validated questionnaires on health related quality of life (EQ5D-5 L) and on medical consumption and productivity losses (iMTA/iPCQ) at 6 and 12 months. Also the health related quality of life 1 month prior to ECLS initiation will be obtained by a questionnaire, if needed provided by relatives. With an estimated overall survival of 62% 210 patients need to be recruited to make a statement on cost effectiveness for all ECLS indications.

**Discussion:**

If our hypothesis that ECLS treatment is cost-effective is confirmed by this prospective study this could lead to an even broader use of ECLS treatment.

**Trial registration:**

The trial is registered at (NCT02837419) registration date July 19, 2016 and with the Dutch trial register, http://www.trialregister.nl/trialreg/admin/rctview.asp?TC=6599

## Background

Extracorporeal life support (ECLS) by means of extracorporeal membrane oxygenation (ECMO) utilization can support the heart and lung for an extended period of time, up to months, an is deployed in the Intensive Care Unit (ICU). The indication for ECLS is acute, potentially reversible cardiac or respiratory failure, when conventional therapy has been inadequate [1]. In addition, ECLS can also be used in patients with terminal respiratory or cardiac failure as a bridge to transplant, and to support cardiopulmonary resuscitation; extracorporeal cardiopulmonary resuscitation.

ECMO is proven beneficial in mature infants with severe but potentially reversible respiratory failure leading to a significantly improved survival without increased risk of severe disability [2]. The increased use of ECLS after the CESAR trial, during the H1N1 pandemic and advances in device technology renewed interest in ECLS utilization in adults [3]. ECLS seems an efficient therapy for cardiac and respiratory failure, but unfortunately no undisputed evidence from randomized controlled trials is delivered yet. Furthermore, currently no detailed information on costs of ECLS therapy is available. The urge for economic evaluations emerges from the increasing deployment of ECLS. The CESAR trial was the first adult trial performed for cost comparison between ECLS and mechanical ventilation for severe adult respiratory distress syndrome (ARDS) [3]. This trial provided data concerning the subgroup of patients with severe ARDS suitable for veno-venous ECLS and compared referral to a center that offered ECLS with conventional management at the original center, not ECLS therapy itself; 68 out of 90 referred patients actually received ECLS therapy. Mean health-care costs per patient were more than twice as high for patients allocated to consideration for ECLS than for those allocated to conventional management. We previously performed a detailed exploratory cost study to gain insight in the hospital costs related to ECLS therapy regardless of indication and found that an ECLS patient carried a mean total hospital cost of €109.407. With a total of 72 patients, our study is the largest economic evaluation, providing a detailed insight of hospital costs in adults treated with ECLS and thus constitutes a first step in assessing it’s cost-effectiveness [4]. The next step in assessing the cost-effectiveness of ECLS therapy is to proceed with an economic evaluation in which costs from a societal perspective are considered. More knowledge about outcome variables such as life years gained and quality adjusted life years (QALY’s) needs to be gathered in a prospective manner. Then, interpretation of the total hospital costs in context to the outcome of ECLS patients is possible with the aim to study whether patients actually benefit from ECLS treatment and at what costs.

A direct comparison between usual care and ECLS, e.g. by means of a randomized trial, is very complex since standard treatment (no ECLS) will result in death in a vast majority of cases. However, several ICU scoring systems exist that predict hospital mortality of individual patients. Depending on the indication, i.e. underlying disease, RESP score, SAVE-score, APACHE-4, SAPS-III or SOFA score are the most reliable for that purpose [5–9].

## Methods/design

The design is a multi-center prospective observational cohort study with the aim to assess the health-related quality of life after ECLS treatment and the associated costs. We expect that survivors of ECLS therapy have a good quality of life and life expectancy. Lifetime predicted costs for QALYs are expected to be cost-effective. Therefore our hypothesis is that ECLS treatment is cost effective.

### Inclusion criteria

All adult patients who receive ECLS will be included. With our consortium we cover nearly all ECLS treatments in The Netherlands. In general, according to the ELSO guidelines, ECLS can be considered in acute severe heart and/or lung failure with high mortality risk despite optimal conventional therapy. ECLS is indicated in most circumstances at 80% mortality risk. Severity of illness and mortality risk is measured as precisely as possible using measurements for the appropriate age group and organ failure.

### Exclusion criteria

Patients in whom the ECLS in only used to bridge a procedure like a high risk percutaneous coronary intervention or during surgery.

### Ethics

The need for ethical approval was waived by the institutional medical ethics board of the University Medical Center of Groningen (protocol number METc 2017/196), and written informed consent will be obtained from all patients or legal representatives.

The predicted mortality for every patient at the initiation of ECLS is used and compared with the actual mortality at one year of each patient. That means that every patient acts as his own control. Depending on the indication or the underlying disease, the APACHE-4, SOFA score, RESP score, or SAVE score will be used to estimate mortality. Although the used models predict hospital mortality instead of one year mortality, the vast majority of mortality is during hospital admission, so the used mortality prediction is expected to be valid for one year survival analyses as well [10, 11].

### Outcome measures

The primary outcome is health related quality of life assessed with the EQ-5D-5 L questioning list 12 months after initiation of ECS treatment.

The EQ-5D-5 L questioning list consists of a descriptive system with 5 domains (mobility, self-care, usual activities, pain/discomfort, anxiety/depression) with 5 possible levels on each which can be converted in to a single summary index score, which represents the societal perspective on quality of life (QoL), and a score on a visual analogue scale, which represents the patient’s perspective in QoL. The iMTA questionnaire is used to assess medical consumption at 6 and 12 months after initiation of ECLS treatment. The iPCQ questionnaire is used to assess productivity losses at 6 and 12 months after initiation of ECLS treatment.

Secondary outcome is one year survival. Additional measurements are EQ-5D-visual analog scale (VAS) score and societal costs. Same parameters are assessed at 6 months, and 1 month before ECLS treatment, the latter retrospectively provided at admission, if needed by relatives.

#### Subgroup analysis

In order to estimate cost-efficacy of ECLS based on indication patients will be categorized into six different subgroups: respiratory – bridge to recovery, respiratory – bridge to transplant, cardiac – bridge to recovery, cardiac – bridge to transplant, cardiac – post-cardiotomy, and extracorporeal cardiopulmonary resuscitation.

#### Sample size calculation

Sample size calculation is based on survival with a good health related-QoL assessed with the EQ-5D-VAS questionnaire one year after initiation of ECLS treatment. Predicted mortality in usual care is 80%. Overall, expected mortality in patients with ECLS treatment is 38%. The increase in survival is thus 42%. To demonstrate an effect on survival 25 patients are needed: risk ratio 0.48 (95%CI 0.28–0.82). In usual care 90% will have a poor outcome, for sake of power analysis defined as dead or an EQ-5D-VAS below the median. With ECLS treatment this will be 69% so 132 patients are needed to demonstrate an effect on QoL. To compensate for a 5% less effect and 5% drop-outs 210 patients will be enrolled to include 200 patients with complete follow-up in the final analysis (RR 0.82; 95%CI 0.75–0.90). Based on a 38% mortality and a study duration of 3 years, 124 (62% out of 200) patients have a complete one year follow-up at the end of the study.

#### Statistics

With the additional information on costs (apart from direct hospital costs already available) assessed within this study it is now possible to calculate the costs of ECLS therapy related to mortality. Based on case-fatality and health related-QoL we will be able to assess QALY’s. For this calculation the predicted mortality at initiation of ECLS therapy is used for comparison. Many patients treated with ECLS survive only after lung transplantation, heart transplantation or implantation of a left ventricular assist device. Life expectancy of the study population can thus be based on the average expectations of that particular patient category. The remaining patients who were treated with ECLS as a bridge to recovery have a reasonable good quality of life [10, 12]. Based on our follow-up study of ICU patients we expect that the median health related-QoL in surviving patients will be 0.83 after one year, which results in 0.36 QALY gained per treated patient. Although additional total healthcare costs and loss of income will be substantial and assumed to exceed €172,200 after one year compared with usual care, lifetime-predicted costs and QALYs is expected to be cost-effective due to the relatively good life-expectancy of survivors. With data from 200 patients of which 62% survive until one-year follow-up we will be able to make a statement about cost-effectiveness for all indications for ECLS.

A cost effectiveness analysis as well as a Budget Impact Analysis will be performed in accordance with the Dutch guidelines [13, 14].

#### Cost effectiveness analysis (CEA)

In the economic evaluation, the primary aim will be to estimate societal costs of ECLS utilization. The secondary aim will be to estimate the cost effectiveness and cost utility of ECLS utilization compared to usual care (from a societal perspective). The cost effectiveness analysis will be performed based on survival. The costs utility analysis will be performed based on EuroQol (EQ-5D-5 L) defined utilities. Results of the cost utility analysis will display the extra costs or savings of ECLS, in order to gain one QALY, compared to usual care. The time horizon of this clinical study will be 1 year, therefore the analysis will not include discounting of costs and effects. Scenario analyses are planned within the trial based evaluation as well as in the model that estimated the effects over a life time horizon. Bootstrap re-sampling will be performed on the cost and effect pairs in order to calculate confidence intervals. Furthermore, cost effectiveness acceptability curves will be plotted, to estimate the probability that ECLS is more cost-effective than usual care, for different amounts of money that a decision maker may be willing to pay for one additional QALY. The trial based results of the CEA, together with available data from literature, will be extrapolated to a life time horizon, using decision modeling in a secondary analysis.

#### Cost analysis

All direct medical cost items, expected to be affected by the ECLS therapy will be registered on a patient level, and valued according to the Dutch standard guidelines for economic evaluations [14]. Non-medical costs such as transportation to an ECLS center, informal care and productivity losses/regained productivity (of patient and partner) will be measured by means of a question in the questionnaire. Costs of resource use outside the hospital, such as productivity losses or traveling expenses will be collected using the relevant parts of the iMTA cost questionnaires.

#### Patient outcome analysis

Utility will be measured using the EuroQol (EQ-5D-5 L) at six months and one year. In order to value the EQ-5D profiles, the new Dutch algorithm will be used [15]. The use of the EQ-5D as a generic measure in economic evaluations facilitates the comparability of (cost effectiveness) results across different studies and interventions.

#### Budget impact analysis (BIA)

Budget impact analysis will be performed from a health care perspective, and in addition from health insurance perspective. The base care scenario will focus on 1 year impact on a national level. Scenario analysis will adopt a 5 to 10 year horizon (life time).

Cost-analysis: A BIA will be conducted to inform decision-makers about the financial consequences of the adoption and diffusion of the use of ECLS in the Dutch healthcare system. The BIA will use a deterministic model. The model input parameters will mainly be based on results of the recently conducted cost study (hospital perspective), the current clinical and cost-effectiveness study, and available literature. The analyses will be conducted from various perspectives, including a direct medical perspective, a budgetary care perspective and a health insurers perspective. For the analysis from a direct medical perspective, only cost within the healthcare sector will be taken into account. Unit prices will be based on Dutch standard prices. For the other perspectives, national tariffs will be applied and the scope of included costs will be limited to those relevant for the perspective concerned. The precision of costs will be in accordance with the described perspectives (€M).

Given the fact that for the populations under study, the standard care consists of ‘no ECLS’, the model will only take changes in the availability and adoption of the use of ECLS into account by calculating the financial consequences of the following scenarios:

- Situation in which all patients that are in principal suitable to use receive ECLS treatment, will receive standard care in the hospital, according to the current practice, which means no ECLS
- Situation in which all suitable patients receive ECLS according to the current study protocol
- Situation in which ECLS utilization will gradually be implemented in new hospitals.
- Situation in which ECLS is considered suitable and available for varying percentages of the targeted population
- Situation in which the indication for ECLS will gradually be broadened.

For each of the presented scenarios, a time horizon up to 5–10 years will be applied. Alternative time horizons will be addressed in sensitivity analyses. Costs will be calculated based on changes in resource use, valued against the price level relevant from the various perspectives. Discounting of future costs will not be applied in the BIA. The model will allow additional analyses for subgroups of patients. These analyses mainly include the shift in the subgroups of patients based on indication and severity. Budget information for relevant subgroups will be made available for decision makers. The planned sensitivity analysis will address the main input parameters and assumptions of the model, and financial consequences of variations in model parameters will be calculated for each of the applied perspectives [16].

## Discussion

The study uses a prospective observational design and not of a randomized controlled trial. A randomized controlled trial in ECLS would be very challenging for both the logistic and ethical reasons. We expect in the current time that the initiation of a randomized controlled trial would lead to bias and therefore impairing the external validity of the results. Our observational design with the use of predicted mortality of individual patients at the moment of initiating of ECLS as an internal comparison resembles the methods of a randomized controlled trial the most. In contrast to a randomized controlled trial our design prevents bias on the basis of indication and selection because all consecutive patients will be included. This guarantees the inclusion of a real world patient population that is clinical relevant.

ECLS is currently offered in many centers worldwide, but application per population range enormously even within countries. It is estimated that in some regions the amount of eligible patients is 10-fold the current number. Although assumed to be beneficial in terms of life saving application may be hampered by the apparent high costs and that ECLS is not covered by the health insurance in some countries. In summary, we expect that ECLS treatment is cost-effectiveness, in this case defined as having an additional benefit worth the additional cost. When cost-effectiveness is made plausible, a gradual increase in ECLS therapy is expected from which many patients will benefit.

### Trial status

The trial is registered at https://clinicaltrials.gov/ (NCT02837419), registration date July 19, 2016.

The trial is registered at the Dutch Trial Register at July 27 2017 http://www.trialregister.nl/trialreg/admin/rctview.asp?TC=6599

Recruitment will start at December 2017, recruiting during 18–24 months, with a 1 year follow up.

## Abbreviations

ARDS: Adult respiratory distress syndrome
ECLS: Extracorporeal life support
ECMO: Extracorporeal membrane oxygenation
ICU: Intensive Care Unit
QALY: Quality adjusted life year
QoL: Quality of life
VAS: Visual analogue scale
